# *Bacillus methylotrophicus* Strain NKG-1, Isolated from Changbai Mountain, China, Has Potential Applications as a Biofertilizer or Biocontrol Agent

**DOI:** 10.1371/journal.pone.0166079

**Published:** 2016-11-10

**Authors:** Beibei Ge, Binghua Liu, Thinn Thinn Nwet, Wenjun Zhao, Liming Shi, Kecheng Zhang

**Affiliations:** 1 State Key Laboratory of Biology of Plant Diseases and Insect Pests, Institute of Plant Protection, Chinese Academy of Agricultural Sciences, Beijing, PR China; 2 Department of Biotechnology, Kyaukse Technological University, Kyaukse, Mandalay, Myanmar; National University of Ireland—Galway, IRELAND

## Abstract

Chemical pesticides are widely used in agriculture, which endangers both environmental health and food safety. Biocontrol is an environmentally-friendly and cost-effective green technique in environmental protection and agricultural production; it generally uses selected bioresources, including beneficial microorganisms. We isolated a novel bacterial strain (NKG-1) from the rare dormant volcanic soils of Changbai Mountain in China’s Jilin Province. The strain was identified as *Bacillus methylotrophicus* using morphological, biochemical, physiological, and phylogenetic 16S rDNA sequencing data. This strain was able to suppress mycelial growth and conidial germination of numerous plant pathogenic fungi on solid media. A greenhouse experiment showed that application of NKG-1 fermentation broth prior to inoculation of *Botrytis cinerea*, the cause of gray tomato mold, inhibited growth of the mold by 60%. Furthermore, application of a 100× dilution of NKG-1 fermentation broth to tomato seedlings yielded a significant increase in seedling fresh weight (27.4%), seedling length (12.5%), and root length (57.7%) compared to the control. When the same dosage was applied in the field, we observed increases in tomato plant height (14.7%), stem diameter (12.7%), crown width (16.3%), and maximum fruit diameter (11.5%). These results suggest that NKG-1 has potential commercial application as a biofertilizer or biocontrol agent.

## Introduction

Over the past decade, chemical fungicides have frequently been used in agriculture; this has resulted in both environmental pollution and food security issues [1]. Intensive use of chemical fungicides has led to fungicide resistance and increased soil contamination; furthermore, these compounds may be toxic to microbial communities [2, 3]. Biocontrol with beneficial microorganisms has been shown to be an environmentally sound option to increase crop yields and prevent outbreaks of pathogenic bacteria [4, 5]. Plant growth-promoting rhizobacteria (PGPR), soil bacteria that colonize plant roots, interact with plant roots and can influence plant growth by scavenging reactive oxygen species, hydrogen peroxide, and volatile compounds [6, 7]. In general, these bacteria inhibit pathogen growth via exudation of antibiotics, inhibition of phytopathogens, induced systemic resistance, parasitism of phytopathogens, and out-competing phytopathogens for space and nutrients [8, 9].

*Bacillus* sp., *Streptomyces* sp., *Penicillium* sp., and *Trichoderma* sp. have been developed as effective microbial control agents that are alternatives to chemical compounds [8, 10, 11–13], and have been tested against various pathogens. *Bacillus* sp. are considered to be safe microorganisms and synthesize substances that suppress many plant pathogens including *Sclerotinia sclerotiorum* [14], *Fusarium oxysporum* [15], *Verticillium* sp. [16], *Eutypa lata* [17], *Botrytis cinerea* [18], *Penicillium digitatum* [19] and *Rhizoctonia solani* [20], both in vitro and in vivo. Previous reports have shown that many different strains of *Bacillus methylotrophicus* have antimicrobial properties, such as the H8 (antagonistic activity towards *Ralstonia solanacearum* [21]) and the BC79 (antagonistic activity towards *Magnaporthe oryzae* and *Bortytis cinerea* [22]) strains. Meanwhile, other strains can produce biosurfactants and ferulic acid degrading products [23, 24].

In this study, we isolated *Bacillus methylotrophicus* NKG-1 and evaluated its ability for biocontrol in an agricultural setting. Our results show that this strain is an effective biocontrol agent against 13 kinds of soil-borne phytopathogenic fungi, including *Botryosphaeria dothidea*, *Phyllosticta ampelicide*, *Valsa ceratosperma*, and *Botrytis cinerea*, and can promote growth of tomato plants in both greenhouse and field settings.

## Materials and Methods

### Sample collection and isolation

Soil samples were collected in the Changbai Mountains (40°15’N, 100°13’E, 2749 m elevation) in China’s Jilin Province. Soil samples were random collected from the top layer (2–15 cm) of soil near the roots of *Pinus koraiensis* plants. Each sample was placed in plastic bag during sample collection, and 100 g were taken from each sample for isolation and stored at 4°C in suitable plastic cans until use. Soil suspensions were prepared (1 g soil in 100 mL sterile water, shaken at 220 rpm for 5 min), serially diluted (down to 10^−6^), and 0.25 mL of supernatant from the last three dilutions was plated on Tryptone-Soybean-Agar (TSA). Agar medium plates were incubated at 28°C, and colonies appeared after 24–48 h. Single colonies were picked and inoculated in Luria-Bertani (LB), Potato-Dextrose-Agar (PDA), and Nutrient-Agar (NA) test tubes. For long term preservation, the isolated strains were stored at -20°C in Eppendorf tubes containing LB and 20% glycerol.

### Morphological, physiological, and biochemical characteristics of NKG-1

The morphological, biochemical, and physiological properties of strain NKG-1 were determined according to the procedures outlined in Bergey’s Manual of Determinative Bacteriology [25]. Standard protocols [26] were used to assess oxidase activity, degradation of gelatin, nitrate reduction, carbon source utilization, and H_2_S production from thiosulfate [27].

### 16S rDNA sequencing and phylogenetic analysis

The DNA of NKG-1 was extracted and purified with a commercial DNA extraction kit according to the manufacturer’s instruction (TransGen Biotech, China). The extracted DNA was amplified (16S rDNA gene) by PCR using the of universal forward primer F1 (5'-GCAGTCGAGCGGACAGAT-3') and reverse primer R1 (5'-AAGGAGGTGATCCAGCCGCA-3'). The PCR mixture contained 2.0 μL 10 × Taq buffer, 1.6 mL MgCl_2_ (25 mM), 1.6 mL dNTP (2.5 mM), 1.0 mL of each primer, 0.5 mL DNA template, 0.2mL Taq DNA polymerase (10000 U mL^-1^), and water to 20μL. The thermocycling conditions were: 1 cycle of 5 min at 94°C; 35 cycles of 30 s at 94°C, 30 s at 51°C, and 1 min 30s at 72°C, and; a final extension step of 10 min at 72°C. PCR products were then sequenced using an automated DNA sequencer (ABI PRISMTM 3730XL DNA Analyzer). The resulting 16S rRNA gene sequences were compared in a BLAST search to the NCBI database. Phylogenetic analysis was performed using the MEGA software package (Version 5.0) [28]. The relationships between sequences were analyzed using the neighbor-joining method. Bootstrap analysis was used to evaluate the tree topology of the neighbor-joining data by analyzing 1,000 randomized data sets.

### Inoculum and fermentation

NKG-1 spores (1.0×10^7^ CFU/ml) were inoculated into 50 mL of LB medium in a 250 ml baffled flask and incubated for 18 h at 25–30°C in a rotary shaker with stirring at 180–200 rpm. Fermentation was performed in 1000 mL Erlenmeyer flasks containing 200 mL of sterile production medium. The fermentation medium was inoculated with 1% (v/v) of a 24 h culture, which consisted of 1.0% peptone, 1.0% sodium chloride, and 1.0% yeast extract at pH 7.2. The inoculated flasks were kept on a rotary shaker (180–200 rpm) with aeration at 25–30°C. The final fermentation product was 10^6^−10^9^ CFU /mL.

### In vitro assay of antagonistic activity

The plate confrontation method was used to detect strains with antagonistic abilities. Fungal cakes were placed in the center of PDA plates, bacterial colonies were cultured 2–3 cm from the cakes at 30°C for 72 h, and inhibition diameter was calculated. Negative control plates had no bacteria (only distilled water). The inhibition effect of culture fermentation of antagonistic bacterium (incubated at 30°C and 220 rpm for 48 h) was detected by the diameter assay [29]. Each treatment was repeated three times. The inhibition rate was calculated as: (control diameter–inhibition diameter) / control diameter × 100%.

*Rhodotorula rubra* was incubated in PDA medium overnight at 28°C and used as an indicator strain in bioassays. Thirteen fungal plant pathogens were tested: *Botrytis cinerea*, *Fulvia fulva*, *Fusarium graminearum*, *Rhizoctonia cerealis*, *Bipolaris maydis*, *Valsa ceratosperma*, *Fusarium oxysporum*, *Colletotrichum lagenarium*, *Pyricularia oryzae*, *Gloeosporium capsici*, *Alternaria alternate*, *Botryosphaeria dothidea*, and *Phyllosticta ampelicide* (all from the Plant Protection Institute of the Chinese Academy of Agricultural Sciences). The fungi were stored at 0–4°C on PDA medium and grown in culture medium at 28°C for 5–7 days before performing the antagonistic experiments.

### Evaluation of isolates in a greenhouse setting

#### Inoculation of *Botrytis cinerea*

Spot experiments were conducted on detached tomato leaves. Healthy leaves were detached from tomato plants (*Solanum lycopersicon* cv. No. 6 Zhongshu) at an early stage. Tomato leaves were inoculated with *Botrytis cinerea* after being sprayed with NKG-1 fermentation broth. Each plant was sprayed with 20 mL of 1.0×10^7^ CFU/mL culture broth. Leaves were placed with the adaxial surface facing up on moist paper towels in an enamel tray. Mycelial plugs of *Botrytis cinerea* removed from 2 d old PDA cultures were individually placed next to the main leaf veins. Detached tomato leaves inoculated with plain PDA were used as a control. The trays with the Botrytis-inoculated leaves were individually covered with a 5.0 mm thick transparent plastic film and incubated at 25°C in a growth chamber (12 h light/12 h dark cycles) for 7 d. Each treatment was repeated three times with ten leaves.

#### Disease assessment

The diameter of the leaf lesion formed around each inoculated mycelial plug was measured, and the disease incidence (%) for each treatment was calculated based as the number of the inoculation points with lesion formation divided by the total number of inoculated plugs. The biocontrol efficacy was rated using the method described by Xue [30]. Each treatment was repeated three times.

### Evaluating the capability for tomato plant fertilization under greenhouse and field conditions

To determine whether or not the potential selected antagonistic isolates could enhance plant growth, we tested the plant growth promotion activity in both greenhouse and field conditions. Fermentation broth (1.0×10^7^ CFU/mL) was irrigated to the roots of plants and also sprayed on the tomato seedlings when they had 2–3 leaves. In total, this occurred three times over ten days. At the end of the experiment, the part of plants that were above ground were removed from the soil, and the fresh weight, dry weight, and root length were measured for fifty greenhouse plants (treatment and control groups). In the field, the plant height, plant width, stem diameter, and fruit size of thirty plants was measured (treatment and control groups). The plant growth promotion efficacy was rated using the method described by Abdallah [31]. Each treatment was repeated three times.

### Statistical analysis

The data were analyzed using analysis of variance (ANOVA) in SPSS 13.0 (SPSS Institute, Chicago, IL). Significant differences between means were compared using Fisher’s protected LSD test at P = 0.05. Differences were considered significant for P < 0.05.

### Ethics Statement

No specific permissions were required to use the locations in this study. We confirm that the location is not privately-owned or protected in any way. No specific permits were required for the field studies described. We confirm that the field studies did not involve endangered or protected species.

## Results

### Identification and characterization of NKG-1

We isolated six bacterial strains that colonized with clear zones. Among those six strains, NKG-1 had the highest antimicrobial activity against *Rhodotorula rubra*; therefore, we focused further investigation on NKG-1.

**Fig 1:**
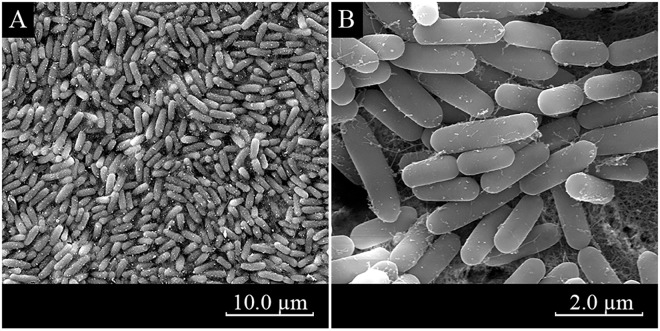
Scanning electron micrographs of *B*. *methylotrophicus* strain NKG-1. (A) Gram-positive staining of NKG-1; scale bar 10.0 μm; (B) Gram-positive staining of NKG-1, scale bar 2.0 μm.

**Fig 2:**
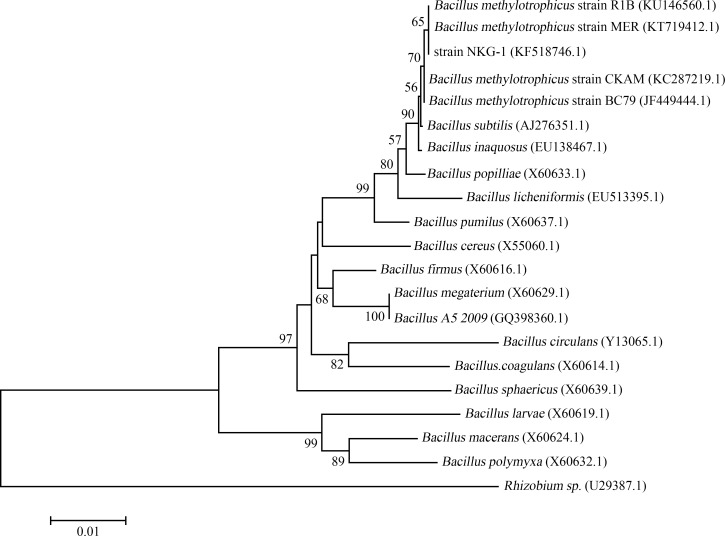
Phylogenetic tree based on 16S rDNA gene sequences from related species of the genus *Bacillus* constructed using the neighbor-joining method with 1,000 bootstrap replicates. Branch length is indicated at each node.

We first studied the morphological characteristics of NKG-1. NKG-1 colonies on LB plates were thin flat, white, opaque, and round with smooth edges. The strain is Gram-positive and scanning electron microscopy showed short rod-like morphology with cells 0.6–0.9 × 2.1–3.2 μm (Fig 1). Colonies were smooth on LB medium and wrinkled on NA or PDA medium. NKG-1 was positive for V-P detection, starch hydrolysis, and esculin hydrolysis. The strain could utilize citrate, urea, tyrosine, xylose, dextrose, glucose, fructose, mannitol, and galactose (Table 1). NKG-1 was characterized taxonomically using both physiological and biochemical tests; all tests indicated that NKG-1 is closely related to *Bacillus methylotrophicus*. To further classify NKG-1, we sequenced part of its 16S rDNA sequence. We obtained a 1347-bp amplification fragment of 16s rDNA by PCR (GenBank accession number KX518746). We compared this sequence with others in the GenBank database, aligned the 16S rDNA sequences with several *Bacillus* sp. strains, and constructed a phylogenetic tree (Fig 2). The results were consistent with those based on morphological, physiological, and biochemical characteristics. The phylogenetic tree clearly showed that strain NKG-1 belongs to the *Bacillus methylotrophicus* branch and is similar to strains CKAM, BC79, MER, and R1B with 99.77%, 95.86%, 95.13%, and 94.33% similarities, respectively.

**Table 1:** Morphological, biochemical, and physiological characterization of NKG-1.

Physiochemical indices	Characteristics		
Morphological characteristics:			
Colony Morphology	thin flat, white color, opaque, round, smooth edge		
Gram stain	+		
Spore shape/position	short rod-like /central		
Physiological characteristics:			
Growth in NaCl at concentration (2.5–8.5%)	+		
Growth at different temperature (20–40°C)	+		
Growth at different pH (5.5–9.5)	+		
Biochemical tests:			
Indole production	_	Arabinose	_
Methyl red	_	Xylose	+
Hydrogen sulfide production	+	Glucose	+
Nitrate reduction	+	Dextrose	+
Starch hydrolysis	+	Fructose	+
Citrate utilization	+	Trehalose	_
Urea utilization	+	Galactose	_
Tyrosine utilization	+	Lactose	+
Voges Proskauer (V-P) detection	+	Glycerol	+
Mannitol	+	—	—

### In vitro screening of antagonistic bacterial activity

The results show that NKG-1 fermentation could inhibit the growth of *R*. *rubra* with an inhibition diameter of 34 mm. To further test the antagonistic activity of NKG-1, we subjected 13 fungal pathogens to plate confrontation tests (S1 Fig). NKG-1 showed good inhibition (>80%) towards *Botryosphaeria dothidea*, *Phyllosticta ampelicide*, *Valsa ceratosperma*, and *Botrytis cinerea* (inhibition zone diameters of 5.7 mm, 5.7 mm, 6.0 mm, and 7.3mm, respectively; Table 2). NKG-1 also inhibited *Pyricularia oryzae*, *Gloeosporium capsici*, *Fusarium graminearum*, and *Colletotrichum lagenarium* by >70%. In general, NKG-1 showed strong antagonistic activity across a broad spectrum (Table 2).

**Table 2:** Inhibition diameter of NKG-1 against 13 pathogens in vitro.

Pathogen	Control diameter (mm)	Inhibition diameter (mm)	Inhibitionratio (%)
*Botryosphaeria dothidea*	85	5.7 ± 0.58	93.33 ± 0.68^a^
*Phyllosticta ampelicide*	78	5.7 ± 0.58	92.74 ± 0.74^a^
*Valsa ceratosperma*	75	6.0 ± 0.01	92.00 ± 0.60^a^
*Botrytis cinerea*	40	7.3 ± 0.58	81.67 ± 1.44^b^
*Pyricularia oryzae*	48	9.7 ± 0.12	79.86 ± 2.41^bc^
*Gloeosporium capsici*	46	11.0 ± 2.65	76.09 ± 5.75^cd^
*Fusarium graminearum*	63	15.3 ± 1.15	75.66 ± 1.84^cd^
*Colletotrichum lagenarium*	29	8.0 ± 1.00	72.41 ± 3.45^d^
*Fulvia fulva*	32	1.20 ± 1.00	62.35 ± 3.13^e^
*Alternaria alternata*.	29	11.0 ± 0.02	62.07 ± 0.40^e^
*Rhizoctonia cerealis*	35	13.7 ± 0.58	60.95 ± 1.65^e^
*Fusarium oxysporum*	47	15.3 ± 1.15	54.90 ± 3.39^f^
*Bipolaris maydis*	43	20.0 ± 1.00	53.49 ± 2.33^f^

Values are the mean of three replicates. Mean ± standard error of the mean (SEM) followed by the same letters in each column are not significantly different (P < 0.05) based on Fisher’s protected least significance test.

### Capability of NKG-1 for disease control in a greenhouse setting

**Fig 3:**
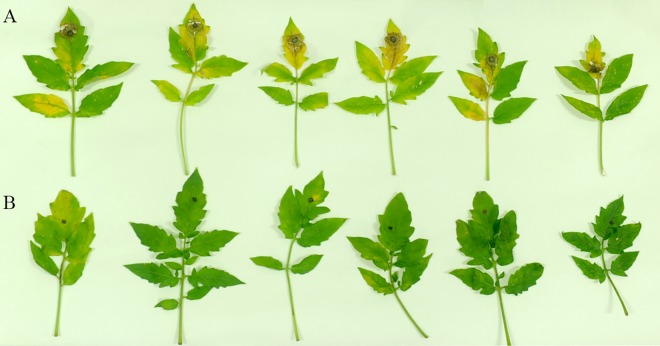
Test of *Botrytis cinerea* pathogenicity on detached tomato leaves over 5 days. (A) tomato leaves were inoculated with *Botrytis cinerea* and incubated at 25°C as a control; (B) tomato leaves were inoculated with *Botrytis cinerea* after being sprayed with *B*. *methylotrophicus* NKG-1 fermentation broth (1.0×10^7^ CFU/mL, from 25°C culture).

We evaluated the efficiency of NKG-1 for controlling *Botrytis cinerea* when applied as a preventive spray. We observed a difference in virulence between the control and treatment groups (tomatoes) in both experiments. Results from the mycelial inoculation experiment (25°C, 3 days) revealed *Botrytis cinerea* infection on tomato leaves. Pathogenicity tests showed that NKG-1 prevented pathogen outbreaks on detached leaves. Control leaves exhibited yellowness and necrotic spots after incubation at 25°C for 3 days; however, treated leaves, which still became yellow, did not have necrotic spots after incubation at 25°C for 5 days (Fig 3). Plant disease was lower in the treatment group (40%) than the control group (100%); thus, NKG-1 decreased *Botrytis cinerea* infection by 60%. On infected leaves, the average lesion diameter was 1.38 ± 0.171 mm in the control group and 0.78 ± 0.155 mm in the treatment group; this difference was statistically significant (*P*<0.05). We can conclude that NKG-1 is effective at controlling *Botrytis cinerea* infection in the field; this is consistent with in vitro results.

### NKG-1 promotes the growth of tomato plants

**Fig 4:**
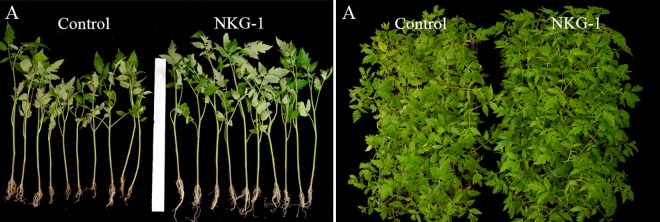
Effect of *B*. *methylotrophicus* NKG-1 fermentation broth on the development of roots and whole tomato seedlings in a greenhouse with five irrigation events during 2 months. (A) effect on root development. (B) effect on whole plant development.

**Fig 5:**
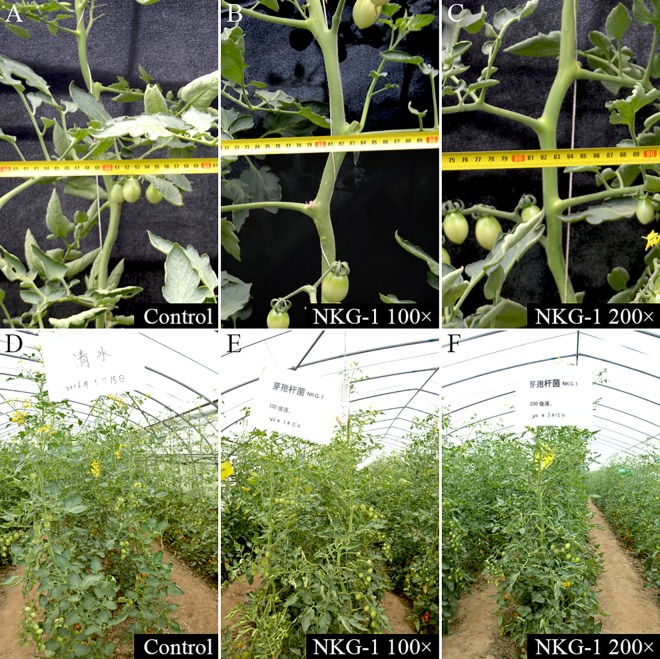
Promotion of tomato plant growth in the field by *B*. *methylotrophicus* strain NKG-1. (A) and (D) tomato seeding plants without any treatment over 3 months (control); (B) and (E) tomato seeding plants after root irrigation and spraying with 100× diluted broth over 3 months; and (C) and (F) tomato seeding plants after root irrigation and spraying with 200× diluted broth over 3 months.

The ability of NKG-1 to promote the growth of tomato plants was tested in greenhouse and field conditions. In the greenhouse, root irritation with 100× and 200× NKG-1 fermentation broth yielded plant heights of 28.23 cm and 27.86 cm, respectively; this corresponded to increases of 12.5% and 11%, respectively, compared with the control (25.09 cm) (Fig 4). Increases in root length (57.7%) and fresh weight (27.4%) were also observed with 100× NKG-1 fermentation broth compared with the control (Table 3). Similar results were observed in the field: root irrigation with 100× and 200× NKG-1 fermentation broth yielded plant heights of 227.03 and 217.16 cm, representing increases of 14.7% and 10.15%, respectively, compared with the control (197.90 cm) (Fig 5). Increased stem diameter (12.7%), crown width (16.3%), and maximum fruit diameter (11.5%) were also observed following treatment with 100× NKG-1 fermentation broth compared with the control (Table 3).

**Table 3:** Effect of NKG-1 root irrigation on tomato plant growth in greenhouse and field conditions.

	Greenhouse			Field			
Treatment	Plant height (cm)	Root length (cm)	Plant fresh weight (g)	Plant height (cm)	Stem diameter (cm)	Crown width (cm)	Max diameter of fruits (cm)
Control	25.09 ± 0.53^a^	3.312 ± 0.86^a^	1.46 ± 0.48^b^	197.90 ± 18.53^a^	3.78 ± 0.82^a^	61.47 ± 9.41^a^	3.04 ± 0.34^a^
NKG-1(100×)	28.23 ± 0.32^b^	5.22 ± 0.41^b^	1.86 ± 0.24^b^	227.03 ± 7.49^b^	4.26 ± 0.47^b^	71.49 ± 9.64^b^	3.39 ± 0.18^b^
NKG-1(200×)	27.86 ± 0.65^b^	5.19 ± 0.62^b^	1.84 ± 0.51^b^	217.16 ± 7.00^b^	4.53 ± 0.49^b^	75.57 ± 8.46^b^	3.47 ± 0.23^b^

Data in the table are Mean ± SEM by three replicates. Values followed by different letters in each column are significantly different (P < 0.05) based on Fisher’s protected least significance test.

## Discussion

Different approaches to plant pathogen control have been exhaustively studied. Biological control agents can be an effective approach to plant disease management, and, unlike synthetic compounds that can be toxic to human and other non-target species, have safe active ingredients that can be adsorbed onto soil colloids [32, 33]. *Bacillus* sp. are typical inhabitants of the rhizosphere and are commonly present on the soil surface [34]. Most strains of *Bacillus* sp. that are antagonistic towards fungi have been isolated from soil. *Bacillus* sp. have biocontrol capabilities, which have been extensively studied and involve cell wall degrading enzymes and antibiotics, as well as active plant resistance by production of molecules that are sensed by plants [8, 9].

Substances produced by *Bacillus* sp. effectively limit normal mycelium growth and spore germination. Many strains of *Bacillus* sp. have been applied as biological control products for plant protection in agriculture, particularly *B*. *subtilis* and *B*. *licheniformis* [35, 36]. However, few *B*. *methylotrophicus* strains have been explored for biocontrol. *B*. *methylotrophicus* strain BC79 has shown biocontrol against rice blast [22], and is an effective biocontrol agent for bacterial tomato wilt [21]. The novel strain in our study has a wider range of fungal antagonism, including 13 tested fungal pathogens. There are additional beneficial functions beyond biocontrol, including P-solubilization (CKAM strain) [37], the potential to degrade ferulic acid and impact antioxidant and rhizospheric enzymatic activates (CSY-F1 strain) [24], and the capability for efficient heterotrophic nitrification-aerobic denitrification (L7 strain) [38].

PGPR is an indispensable part of rhizosphere biota that, when grown in association with host plants, can stimulate the growth of the host. Successful rhizobacteria are highly adaptable to a wide variety of environments and typically exhibit the function of plant promotion resulting in faster growth rates than normal plants. These PGPR bacteria are considered a significant factor in agricultural management practices. The potential for PGPR application in agriculture is steadily increasing, as it is an attractive way to potentially replace chemical fertilizers, pesticides, and other supplements [39]. These rhizosphere microorganisms likely produce large quantities of growth-promoting substances that indirectly influence plant morphology. *Pseudomonas fluorescens* and *Bacillus subtilis* strains are the most promising candidates for indirect stimulation [40]. Only the CKAM and H8 strains of *B*. *methylotrophicus* have shown plant growth-promoting properties [22].

This study is the first report regarding the pathogen antagonism and plant growth-promoting potential of *B*. *methylotrophicus* NKG-1, which was isolated from the *P*. *koraiensis* rhizosphere of mountainous volcanic soil. The rhizosphere of healthy pine trees in areas impacted by soil-borne phytopathogenic fungi could provide a feasible source material for isolating microorganisms with antagonistic abilities. We identified NKG-1 using morphological, biochemical, and rDNA gene analyses. The utilization of citrate by NKG-1 distinguishes it from strain BC-79, which cannot utilize citrate [22]. Phylogenetic analysis confirmed the identity of NKG-1; a BLASTn search of GenBank 16S rDNA sequences indicated that NKG-1 belongs to *Bacillus methylotrophicus* and shows similarities to strains CKAM, BC79, MER, and R1B (99.77%, 95.86%, 95.13%, and 94.33%, respectively). Despite the 99.77% similarity to strain CKAM, NKG-1 and CKAM are functionally different. CKAM is mainly used for P-solubilization [37]. The in vitro prescreening test on NKG-1 showed significant antifungal activities against *Botryosphaeria dothidea*, *Phyllosticta ampelicide*, *Valsa ceratosperma*, and *Botrytis cinerea* (>80% inhibition ratio; Table 2). When applied in a preventive spray to control *Botrytis cinerea* on tomato leaves, the biocontrol efficacy was 60% (Fig 3). NKG-1 also promoted growth of tomato seedlings in greenhouse and field conditions (Figs 4 and 5). Although there are many *Bacillus* strains with similar functions (i.e., biocontrol and promotion of plant growth), most do not show these traits in both greenhouse and field conditions; thus, NKG-1 could be a potential candidate for use as a biofertilizer or biocontrol agent.

## Conclusions

NKG-1 was isolated from the rhizosphere of a *P*. *koraiensis* sample from a dormant volcano in southern China. We identified this strain as *Bacillus methylotrophicus* based on morphological, biochemical, physiological, and phylogenetic analyses. NKG-1 shows significant antifungal and fertilization activities when applied to tomato plants. It is one of only a few *B*. *methylotrophicus* strains to display these characteristics in both the greenhouse and the field. Strain NKG-1 could be a potential candidate for use as a biofertilizer or biocontrol agent.

## Supporting Information

S1 Fig Antibacterial effect of *Bacillus methylotrophicus* NKG-1 fermentation broth (1.0×10^7^ CFU/mL) on mycelial growth of different fungi after 7 days. (A) *Rhodotorula rubra*, (B) *Botryosphaeria dothid*, (C) *Phyllosticta ampelicide*, (D) *Valsa ceratosperma*, (E) *Botrytis cinerea*, (F) *Pyricularia oryzae*, (G) *Gloeosporium capsici*, (H) *Fusarium graminearum*, (I) *Colletotrichum lagenarium*, (J) *Fulvia fulva*, (K) *Alternaria alternata*, (M) *Rhizoctonia cerealis*, (N) *Fusarium oxysporum*, and (O) *Bipolaris maydis*.
(TIF)
