# Supplementary figures and images for: Bacillus methylotrophicus Strain NKG-1, Isolated from Changbai Mountain, China, Has Potential Applications as a Biofertilizer or Biocontrol Agent
Source: PLoS One. 2016 Nov 10;11(11):e0166079. doi: 10.1371/journal.pone.0166079 (PMC5104391; doi:10.1371/journal.pone.0166079)

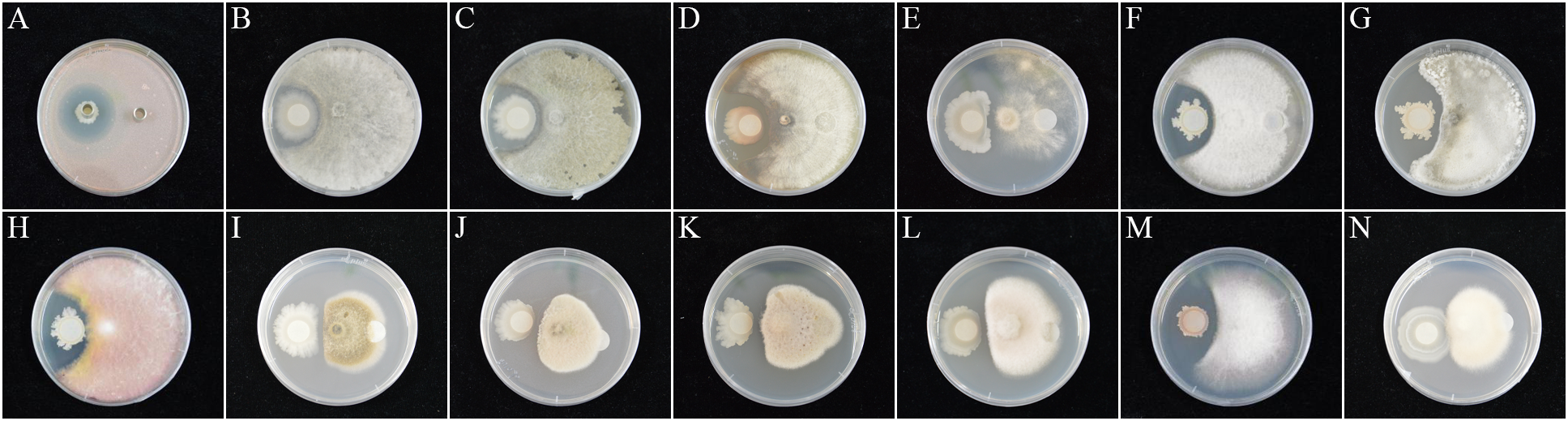

Supplement: S1 Fig — (A) Rhodotorula rubra, (B) Botryosphaeria dothid, (C) Phyllosticta ampelicide, (D) Valsa ceratosperma, (E) Botrytis cinerea, (F) Pyricularia oryzae, (G) Gloeosporium capsici, (H) Fusarium graminearum, (I) Colletotrichum lagenarium, (J) Fulvia fulva, (K) Alternaria alternata, (M) Rhizoctonia cerealis, (N) Fusarium oxysporum, and (O) Bipolaris maydis. (TIF) [file pone.0166079.s001.tif]
